# Conserved FimK Truncation Coincides with Increased Expression of Type 3 Fimbriae and Cultured Bladder Epithelial Cell Association in Klebsiella quasipneumoniae

**DOI:** 10.1128/jb.00172-22

**Published:** 2022-08-25

**Authors:** Sundharamani Venkitapathi, Yalini H. Wijesundara, Samuel A. Cornelius, Fabian C. Herbert, Jeremiah J. Gassensmith, Philippe E. Zimmern, Nicole J. De Nisco

**Affiliations:** a Department of Biological Sciences, The University of Texas at Dallas, Richardson, Texas, USA; b Department of Chemistry and Biochemistry, The University of Texas at Dallas, Richardson, Texas, USA; c Department of Urology, University of Texas Southwestern Medical Center, Dallas, Texas, USA; Geisel School of Medicine at Dartmouth

**Keywords:** *Klebsiella pneumoniae*, *Klebsiella quasipneumoniae*, urothelium, fimbriae, urinary tract infection, *Klebsiella*, virulence regulation

## Abstract

Klebsiella spp. commonly cause both uncomplicated urinary tract infection (UTI) and recurrent UTI (rUTI). Klebsiella quasipneumoniae, a relatively newly defined species of Klebsiella, has been shown to be metabolically distinct from Klebsiella pneumoniae, but its type 1 and type 3 fimbriae have not been studied. K. pneumoniae uses both type 1 and type 3 fimbriae to attach to host epithelial cells. The type 1 fimbrial operon is well conserved between Escherichia coli and K. pneumoniae apart from *fimK*, which is unique to Klebsiella spp. FimK contains an N-terminal DNA binding domain and a C-terminal phosphodiesterase (PDE) domain that has been hypothesized to cross-regulate type 3 fimbriae expression via modulation of cellular levels of cyclic di-GMP. Here, we find that a conserved premature stop codon in *K. quasipneumoniae fimK* results in truncation of the C-terminal PDE domain and that *K quasipneumoniae* strain KqPF9 cultured bladder epithelial cell association and invasion are dependent on type 3 but not type 1 fimbriae. Further, we show that basal expression of both type 1 and type 3 fimbrial operons as well as cultured bladder epithelial cell association is elevated in KqPF9 relative to uropathogenic K. pneumoniae TOP52. Finally, we show that complementation of KqPF9*ΔfimK* with the TOP52 *fimK* allele reduced type 3 fimbrial expression and cultured bladder epithelial cell attachment. Taken together these data suggest that the C-terminal PDE of FimK can modulate type 3 fimbrial expression in K. pneumoniae and its absence in *K. quasipneumoniae* may lead to a loss of type 3 fimbrial cross-regulation.

**IMPORTANCE** *K. quasipneumoniae* is often indicated as the cause of opportunistic infections, including urinary tract infection, which affects >50% of women worldwide. However, the virulence factors of *K. quasipneumoniae* remain uninvestigated. Prior to this work, *K. quasipneumoniae* and K. pneumoniae had only been distinguished phenotypically by metabolic differences. This work contributes to the understanding of *K. quasipneumoniae* by evaluating the contribution of type 1 and type 3 fimbriae, which are critical colonization factors encoded by all Klebsiella spp., to *K. quasipneumoniae* bladder epithelial cell attachment *in vitro.* We observe clear differences in bladder epithelial cell attachment and regulation of type 3 fimbriae between uropathogenic K. pneumoniae and *K. quasipneumoniae* that coincide with a structural difference in the fimbrial regulatory gene *fimK*.

## INTRODUCTION

Klebsiella spp., including K. pneumoniae, *K. variicola*, *and K. quasipneumoniae*, are common causes of both acute and recurrent UTIs (1–5). Recurrent UTI (rUTI), defined as two symptomatic UTI episodes within 6 months or three within 12 months, poses a major health issue with ~50% of UTIs in postmenopausal women estimated to develop into rUTI (6–8). Uropathogenic Escherichia coli (UPEC) is the most common organism implicated in rUTI and is followed in prevalence by the genus Klebsiella, which accounts for 15 to 17% of cases (9, 10). K. pneumoniae has also been reported to be one of the most common causes of hospital-acquired UTI (11, 12). K. pneumoniae and the closely related species *K. quasipneumoniae* and *K. variicola* are a growing clinical concern due to the prevalence of multidrug-resistant strains in patients with both community-acquired and hospital-acquired UTIs (2, 3). Alongside extended-spectrum beta-lactamases (ESBLs), Klebsiella spp. isolated from UTI patients often encode carbapenemases, aminoglycoside-modifying enzymes, and fluoroquinolone resistance genes (13–17).

K. pneumoniae expresses both type 1 and type 3 fimbrial operons (18, 19). These fimbriae have been reported to play a role in attachment and invasion of bladder epithelial cells in a mannose-sensitive and -insensitive fashion, respectively (20–23). Type 1 fimbriae are encoded by the *fim* operon and are expressed among most *Enterobacteriaceae* (24, 25). The *fim* operon of K. pneumoniae shares a high degree of similarity with E. coli (26) except for the presence of *fimK* at the end of the operon (27). The expression of type 1 fimbriae is regulated by the phase transition of the invertible *fimS* regulatory sequence (*fim* switch) (28). The phase variation is mediated by recombinases FimB and FimE, which are also encoded within the *fim* operon (29). Type 3 fimbriae, though initially identified in Klebsiella spp., have also been reported in other *Enterobacteriaceae*, including *Serratia* spp., Enterobacter spp., and *Citrobacter* spp., and more recently encoded within plasmids of a few E. coli isolates (30–34). Type 3 fimbriae are encoded by the *mrk* operon with *mrkA* encoding the major structural subunit and *mrkD* encoding the adhesin (35, 36). The expression of type 3 fimbriae is regulated by the *mrkHIJ* gene cluster (37, 38). MrkH is a PilZ domain-containing protein that functions as the major activator of *mrkABCDF* transcription upon binding to the second messenger cyclic-di-GMP. MrkI has been reported to contain a LuxR type DNA binding domain and functions as a minor activator of *mrkABCDF* (38). MrkJ acts as a phosphodiesterase (PDE) hydrolyzing cyclic di-GMP preventing its binding to MrkH and thereby repressing *mrkABCDF* expression (37).

*K. quasipneumoniae* were previously classified as part of K. pneumoniae phylogroups KpIIA and KpIIB but were relatively recently distinguished from K. pneumoniae as a new species with distinct metabolic phenotypes (39). *K. quasipneumoniae* isolates have been collected from patients with bloodstream infections as well as from the urine of patients with uncomplicated UTI and rUTI (3, 5, 40). However, little is known about how *K. quasipneumoniae* interacts with the bladder environment. In this study, genomic analysis of *K. quasipneumoniae* showed a difference in the structure of the type 1 fimbrial regulatory gene *fimK* compared to K. pneumoniae. The *K. quasipneumoniae fimK* allele is truncated and lacks the C-terminal PDE domain conserved among K. pneumoniae isolates. Because the PDE activity of K. pneumoniae FimK has been hypothesized to cross-regulate type 3 fimbriae by reducing cyclic-di-GMP levels necessary for MrkH activation, we sought to investigate differences in type 3 fimbrial regulation between the two species (41). We show that attachment of the *K. quasipneumoniae* strain KqPF9 to cultured bladder epithelial cells is dependent on type 3 but not type 1 fimbriae. Further, we demonstrate that the *fimK* C-terminal PDE domain downregulates type 3 fimbriae when expressed in both K. pneumoniae and *K. quasipneumoniae* but that *K. quasipneumoniae fimK*, which lacks this domain, does not affect type 3 fimbriae expression. Taken together, this study defines the contribution of type 1 and type 3 fimbriae to *K. quasipneumoniae* bladder epithelial cell association and identifies a possible role for *fimK* in the regulation of type 3 fimbriae in K. pneumoniae but not in *K. quasipneumoniae*.

## RESULTS

### Attachment and invasion of Klebsiella quasipneumoniae to cultured human bladder epithelial cells are mannose insensitive.

**FIG 1:**
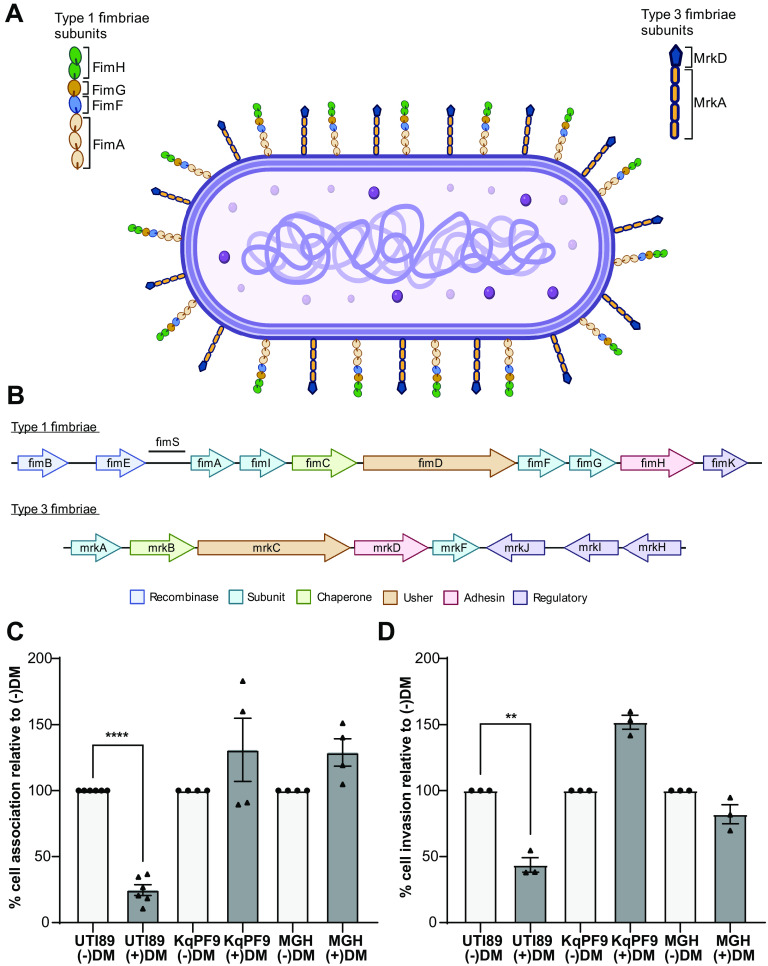
*K. quasipneumoniae* association and invasion to bladder epithelial cells is mannose insensitive. (A) Illustration of Klebsiella type 1 and type 3 fimbriae. FimA and FimH, MrkA, and MrkD represent the major structural subunit and adhesin for type1 and 3 fimbriae, respectively. FimF and FimG represent minor structural subunits of type 1 fimbriae. (B) Schematic of the *fim* and *mrk* operons encoding type 1 and type 3 fimbriae of *K. quasipneumoniae* isolate PF9 (KqPF9), respectively. Illustrations were made using Biorender.com. (C and D) 5637 bladder epithelial cell association and invasion in the absence (−DM) and presence (+DM) of 2.5% d-mannose. Uropathogenic E. coli strain UTI89 was used as mannose-sensitive control, and K. pneumoniae type strain MGH78578 was used as mannose-insensitive control. Bladder epithelial cell association of PBS control was evaluated as 100%, and the effect of mannose was determined relative to respective PBS control. Experiments were performed in biological and technical triplicate. The error bars indicate standard error of mean. Significance was evaluated by two-tailed, paired Student's *t* test. **, *P* < 0.01 and ****, *P* < 0.0005.

Cell association and invasion phenotypes of Klebsiella quasipneumoniae have not been previously reported. We therefore first sought to use KqPF9, a *K. quasipneumoniae* strain that we recently isolated from a postmenopausal woman with rUTI to evaluate *K. quasipneumoniae* cell association and invasion. The complete genome of KqPF9 has been previously reported and contains one chromosome (5.27 Mbp) and four plasmids (of sizes 399,394 bp, 4,730 bp, 4,096 bp, and 4,000 bp) (5). Because analysis of the KqPF9 genome revealed that the chromosome encodes both type 1 (*fim*) and type 3 (*mrk*) fimbrial operons, we first sought to determine if KqPF9 cell association was dependent on mannose-sensitive type 1 fimbriae or mannose-insensitive type 3 fimbriae (Fig. 1A and B) (5, 23, 42, 43). We measured the effect of d-mannose on KqPF9 association with bladder epithelial cell line 5637 (ATCC) via cell association assay using K. pneumoniae 78578 (KpMGH78578; ATCC), which expresses both type 1 and type 3 fimbriae, as a mannose-insensitive control and uropathogenic E. coli UTI89, which expresses only type 1 fimbriae, as a mannose-sensitive control (19, 42, 44, 45). While a significant 75.4% reduction in 5637 bladder epithelial cell association was observed for UTI89 in the presence of d-mannose, KqPF9 and KpMGH78578 showed no significant decrease in association (Fig. 1C and Fig. S1A in the supplemental material). Then, using gentamicin protection assays to measure cultured bladder epithelial cell invasion, we observed that d-mannose treatment similarly reduced invasion frequencies of UTI89 (56.3%), while KqPF9 and KpMGH78578 invasion frequencies were not significantly reduced (Fig. 1D and Fig. S1B). This pattern of invasion is consistent with previous reports in the uropathogenic K. pneumoniae isolate 3091, which expresses both type 1 and type 3 fimbriae (23). To confirm these results in independent association models, we also evaluated KqPF9 cell association by yeast and tanned human red blood cell (RBC) agglutination assays. UTI89 and KqPF9 were able to agglutinate Saccharomyces cerevisiae strain L40, but the addition of d-mannose abrogated yeast agglutination only by UTI89 (Table S1) (43). Similarly, tanned human RBCs, which agglutinate in a type 3 fimbriae-specific manner, were only agglutinated by KqPF9 (Table S1) (25, 30). Together, these results suggest that KqPF9 expresses type 3 fimbriae and that KqPF9 association and invasion of cultured bladder epithelial cells is mannose insensitive and therefore may not rely on type 1 fimbriae.

### Type 3 but not type 1 fimbriae are required for *K. quasipneumoniae* attachment to cultured bladder epithelial cells.

**FIG 2:**
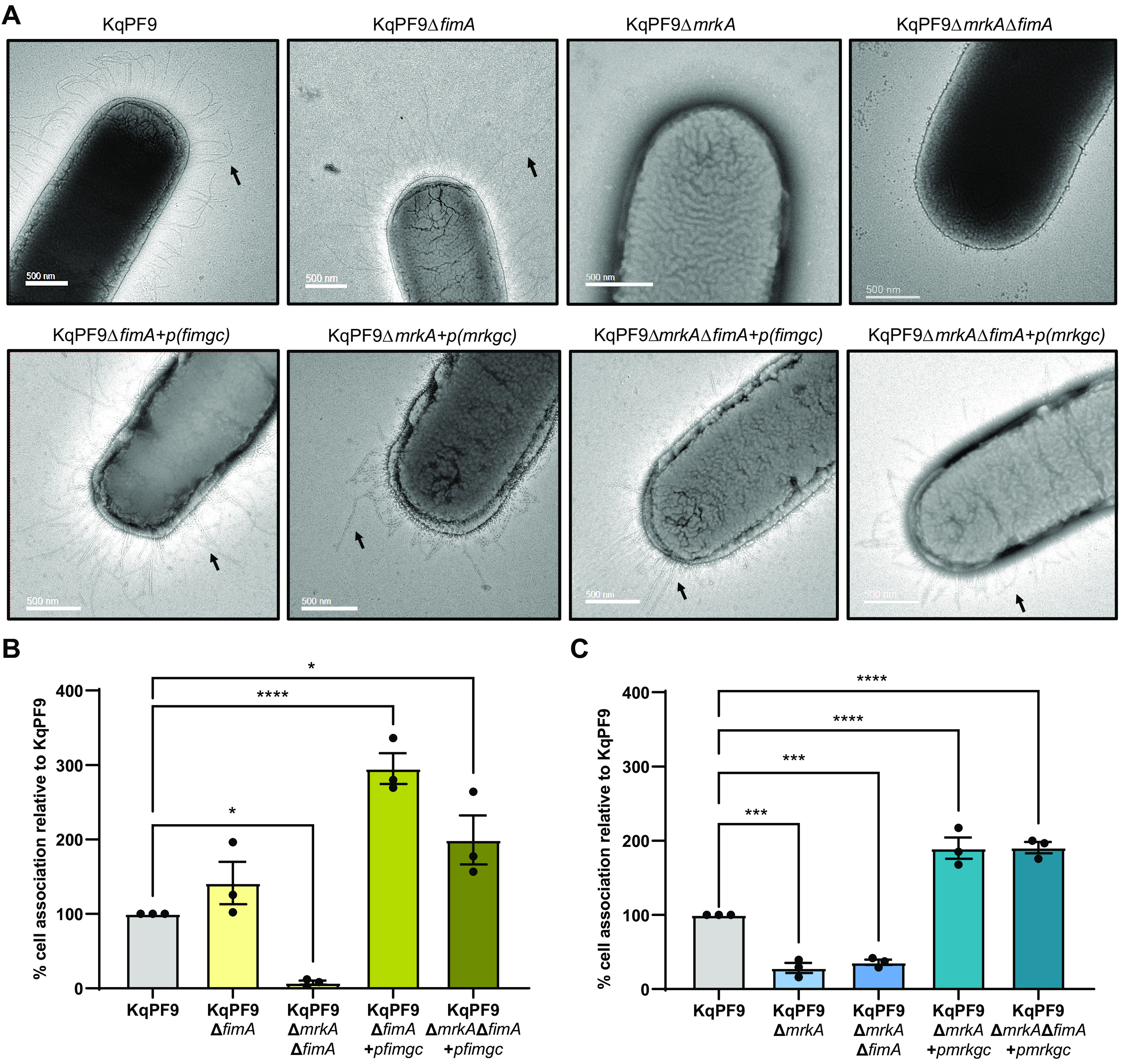
*K. quasipneumoniae* association to cultured bladder epithelial cells is dependent on type 3 fimbriae. (A) Representative electron micrographs of KqPF9 and isogenic KqPF9Δ*fimA*, KqPF9Δ*mrkA*, and KqPF9Δ*fimA*Δ*mrkA* mutant and complement strains. *fimgc* Indicates the *fim* gene cluster (*fimAICDFGHK*), and *mrkgc* indicates the *mrk* gene cluster (*mrkABCDF*). Arrows point to fimbriae. Scale bar = 500 nm. (B and C) Cell association of KqPF9 and isogenic type 1 and type 3 fimbriae mutants and mutants expressing respective complementing plasmids to 5637 bladder epithelial cells. The cell association of respective mutants were evaluated relative to KqPF9. The experiments were performed in biological and technical triplicate. The error bars indicate standard error of mean. Significance was evaluated using one-way ANOVA with Dunnett’s multiple comparisons *post hoc*. *, *P* < 0.05; ***, *P < *0.005; and ****, *P* < 0.0005.

To evaluate the role of type 1 and type 3 fimbriae of KqPF9 in cultured bladder epithelial cell association, we generated strains with targeted deletions of *fimA* and *mrkA*, which encode the major fimbrial subunits of the type 1 and 3 fimbrial operons, respectively (36, 46–48). Gene deletions were confirmed by PCR, and target gene expression was measured in each mutant and complement strain by quantitative reverse transcriptase PCR (qRT-PCR) (Fig. S2A to E). We next used negative stain electron microscopy to evaluate fimbrial structures produced by each mutant. While fimbrial structures were apparent in wild-type KqPF9 and isogenic type 1 fimbriae mutant KqPF9*ΔfimA*, no fimbrial structures were visible in the isogenic type 3 fimbriae mutant KqPF9*ΔmrkA* or double mutant strain KqPF9*ΔmrkAΔfimA* (Fig. 2A). These data suggest that KqPF9 may be preferentially expressing type 3 fimbriae during static growth in Luria Bertani (LB). Further, fimbrial structures were visible in all complemented mutant strains (Fig. 2A). We then performed cell association assays with cultured 5637 bladder epithelial cells to determine the contribution of type 1 and type 3 fimbriae to KqPF9 cell adhesion. The isogenic type 1 fimbriae mutant KqPF9*ΔfimA* strain showed no significant alteration in bladder epithelial cell association relative to wild type (Fig. 2B and Fig. S2F). However, as previously reported in K. pneumoniae, complementation by overexpression of type 1 fimbrial gene cluster (*fimAICDFGHK*) significantly increased bladder epithelial cell association (19). A significant 92.4% decrease in cell association was observed in the double fimbriae mutant KqPF9*ΔmrkAΔfimA*, suggesting that type 3 fimbriae may contribute to bladder epithelial cell association *in vitro* (Fig. 2B). Complementation with type 1 fimbrial gene cluster (*fimAICDFGHK*) was able to rescue the cell association phenotype in KqPF9*ΔmrkAΔfimA* strain, which may be attributed to overexpression of the operon (Fig. S2D). In contrast to KqPF9*ΔfimA*, the type 3 fimbriae mutant KqPF9*ΔmrkA* showed a significant 71.6% reduction in bladder epithelial cell association, which was complemented by overexpression of the type 3 fimbrial gene cluster (*mrkABCDF*) (Fig. 2C and Fig. S2G). The reduction of cell association in KqPF9*ΔmrkA* and the KqPF9*ΔmrkAΔfimA* double fimbriae mutant was also rescued by overexpression of the *mrkABCDF* gene cluster (Fig. 2C). Taken together, these data suggest that *in vitro* association of KqPF9 with bladder epithelial cells is dependent on type 3 fimbriae. However, the ability of ectopic expression of the type 1 fimbrial gene cluster to rescue bladder epithelial cell adhesion in KqPF9*ΔmrkA* suggests that type 1 fimbriae are sufficient for adhesion even in the absence of type 3 fimbriae but may not be expressed endogenously under the conditions tested.

### Elevated fimbrial expression and cultured bladder epithelial cell association in KqPF9 compared to K. pneumoniae TOP52.

**FIG 3:**
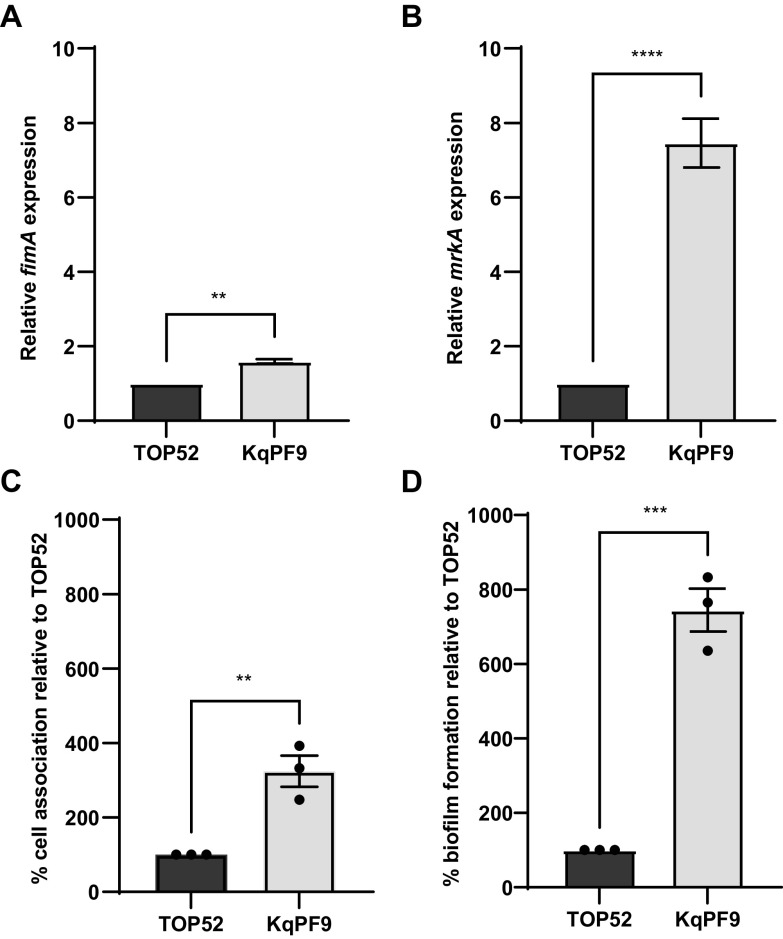
Expression of type 1 and type 3 fimbriae as well as bladder epithelial cell attachment and biofilm formation is elevated in KqPF9 relative to TOP52. (A and B) qRT-PCR analysis of type 1 fimbriae (*fimA*) and type 3 fimbriae (*mrkA*) expression in TOP52 and KqPF9 isolates. The expression values of *fimA* and *mrkA* were normalized to *rho*, and the fold change was determined relative to TOP52. (C) Percent association KqPF9 with 5637 bladder epithelial cells relative to TOP52. (D) Static biofilm formation of TOP52 and KqPF9. Percent KqPF9 biofilm formation was determined relative to TOP52. All experiments were performed at least in biological and technical triplicate. All error bars indicate standard error of mean and two-tailed, paired Student's *t* test was used to evaluate significance. **, *P* < 0.01; ***, *P* < 0.005; and ****, *P* < 0.0005.

Because variation in urovirulence phenotypes has been previously reported in *K. variicola*, which was also recently speciated from K. pneumoniae, we next wanted to directly compare fimbrial expression and cultured bladder epithelial cell association between KqPF9 and the well-studied uropathogenic K. pneumoniae strain TOP52 *in vitro* (2, 27). Quantitative RT-PCR analysis of *fimA* and *mrkA* expression in statically cultured TOP52 and KqPF9 showed a 1.5-fold higher *fimA* expression and 7.4-fold higher *mrkA* expression in KqPF9 relative to TOP52 (Fig. 3A and B). We then compared cell association frequencies between the two strains and observed that relative bladder epithelial association of KqPF9 was 324.1% higher than TOP52 (Fig. 3C and Fig. S3A). Because we observed significantly increased expression of the major type 3 fimbrial subunit *mrkA* in KqPF9, and biofilm formation has been previously attributed to type 3 fimbriae in K. pneumoniae, we also evaluated biofilm formation in each strain (49). In accordance with the elevated *mrkA* expression in KqPF9, we also observed a 744% increase in biofilm formation in KqPF9 with respect to TOP52 (Fig. 3D). Also, since expression of the *fim* operon is controlled by inversion of the *fimS* regulatory element, we performed a *fimS* phase assay and observed that statically grown TOP52 and KqPF9 contained populations both in the “on” and “off” orientation (Fig. S3B and C).

### The type 1 fimbrial regulatory gene *fimK* is truncated in *K. quasipneumoniae*.

**FIG 4:**
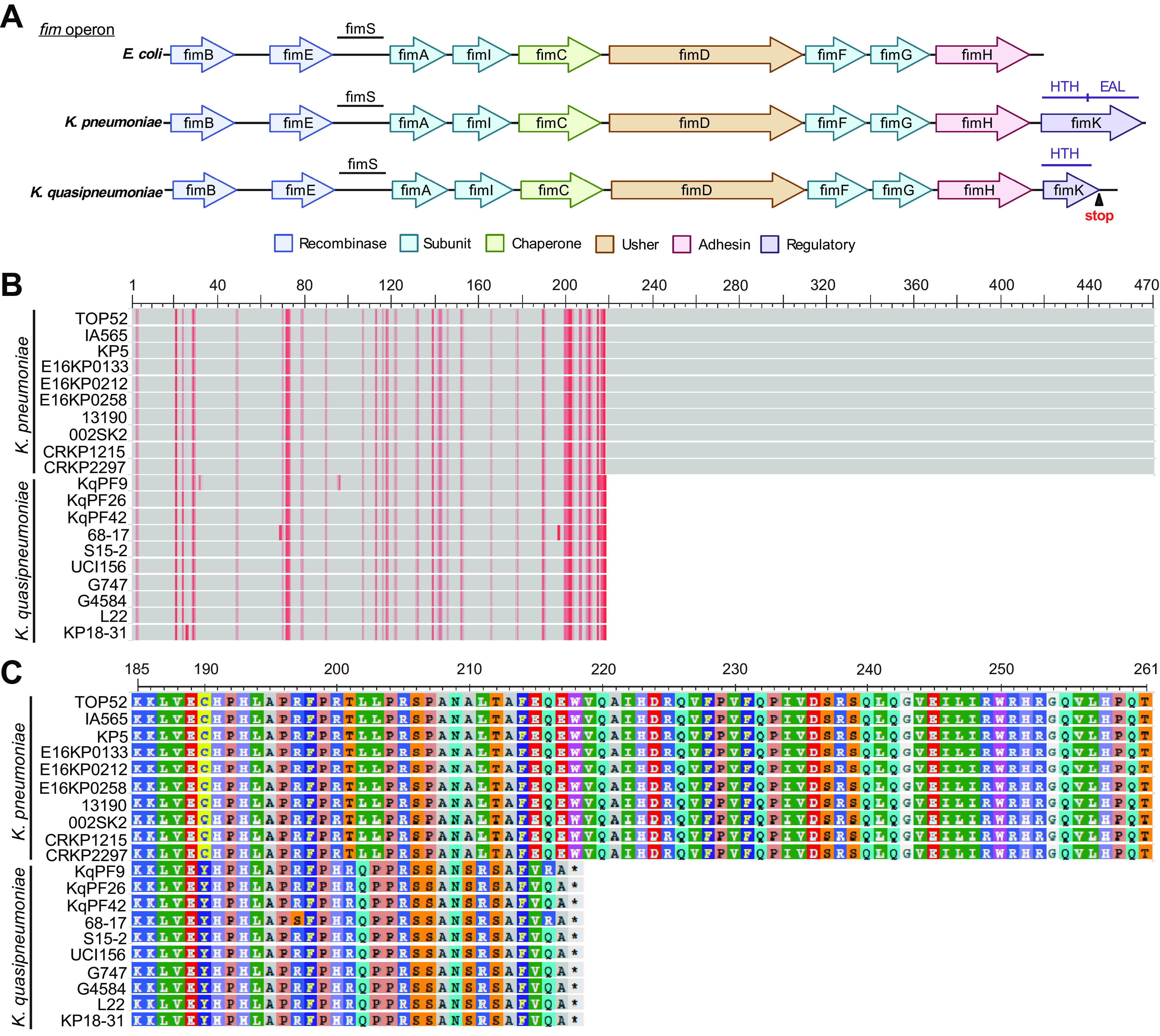
The FimK C-terminal PDE is truncated in *K. quasipneumonaie*. (A) Comparison of the *fim* operon encoding type 1 fimbriae among the Enterobacteriaceae, E. coli, K. pneumoniae, and *K. quasipneumoniae*. E. coli does not encode *fimK*, and *K. quasipneumoniae* encodes a truncated *fimK* encompassing only the putative helix-turn-helix (HTH) domain with a premature stop codon truncating the EAL phosphodiesterase domain. Schematic made using Biorender.com. (B) Amino acid alignment of FimK sequences of K. pneumoniae and *K. quasipneumoniae* strains deposited in NCBI database is shown with respective sequence IDs. The start and end positions indicate the beginning and end of the FimK amino acid sequence. Mismatches are indicated in red. (C) A magnification of the alignment in B encompassing the region between amino acids 185 and 261 showing the truncation of *K. quasipneumoniae* FimK at position 218.

We next used previously generated whole-genome sequences of TOP52 and KqPF9 to identify genotypic differences explaining the increased expression of *mrkA* and bladder epithelial cell association observed in KqPF9 (5, 50, 51). While we observed no differences in *mrk* operon structure between the two Klebsiella species, we found that the type 1 fimbrial regulatory gene *fimK* was truncated in KqPF9 (Fig. 4A). Indeed, multiple sequence alignment of the FimK amino acid sequences extracted from 10 K. pneumoniae and 10 *K. quasipneumoniae* genomes deposited in the NCBI database revealed that the premature stop codon observed in KqPF9 was conserved among *K. quasipneumoniae* isolates resulting in a 218 aa protein compared to the 470-aa protein observed in all K. pneumoniae isolates (Fig. 4B and C) (52, 53). The *fimK* gene of K. pneumoniae encodes an N-terminal helix-turn-helix (HTH) DNA binding domain and a C-terminal phosphodiesterase (PDE) domain (41). The premature stop codon observed in *K. quasipneumoniae* results in complete truncation of the C-terminal PDE domain. Because cyclic-di-GMP regulates the expression of type 3 fimbriae through MrkH, it has been hypothesized that the FimK PDE domain may regulate type 3 fimbrial expression by modulating cyclic-di-GMP levels (38, 41). We, therefore, hypothesized that the elevated *mrkA* expression and bladder epithelial cell association observed in KqPF9 may be due to the absence of the C-terminal PDE domain that is present in K. pneumoniae FimK.

### Role of *mrkH* and *mrkJ* in *K. quasipneumoniae* type 3 fimbriae expression.

**FIG 5:**
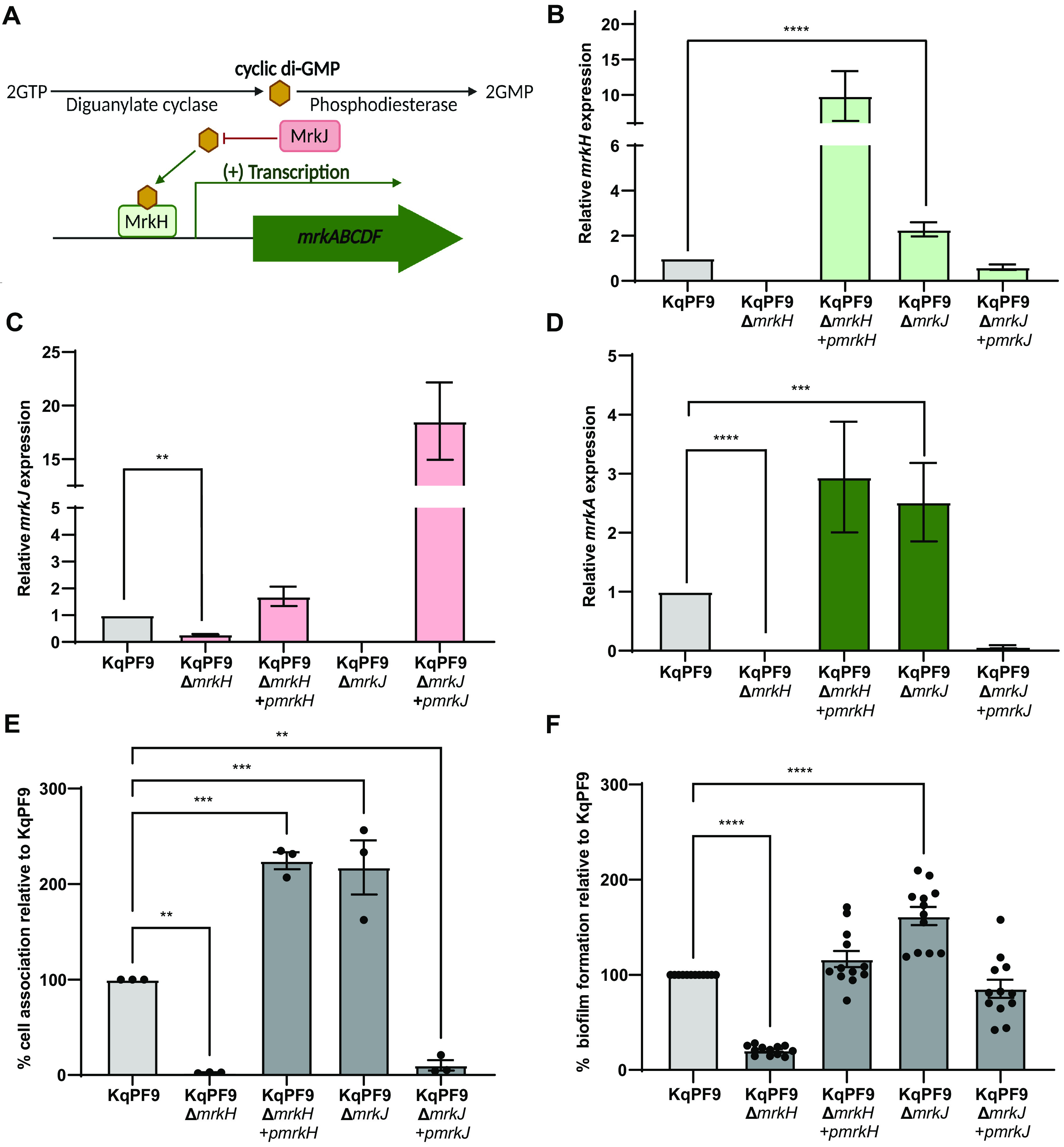
The role of *mrkH* and *mrkJ* in type 3 fimbriae expression is conserved in KqPF9. (A) Schematic of regulation of the *mrk* operon by transcriptional regulator MrkH, phosphodiesterase MrkJ, and cyclic di-GMP in K. pneumoniae. (B to D) qRT-PCR analysis of *mrkH* (B), *mrkJ* (C), and type 3 fimbriae (*mrkA*) (D) expression in KqPF9Δ*mrkH* and Δ*mrkJ* mutant and complement strains. Expression of each gene was normalized to *rho*, and the fold change is expressed relative to wild type. (E) Cell association of KqPF9Δ*mrkH* and Δ*mrkJ* mutant and complemented strains to 5637 bladder epithelial cells. The cell association of respective mutant strains was evaluated relative to wild-type KqPF9. (F) Static biofilm formation of KqPF9 along with isogenic mutants of respective genes, and strains expressing appropriate complementing plasmids. The biofilm formation of the mutant strains was determined relative to KqPF9. All experiments were performed in at least in biological and technical triplicate. The error bars indicate standard error of mean. Statistical testing was performed by one-way ANOVA analysis with Dunnett’s multiple-comparison test. **, *P* < 0.01; ***, *P* < 0.005; and ****, *P* < 0.0005.

Before investigating differences in FimK function between KqPF9 and TOP52, we first sought to determine if known regulators of type 3 fimbrial expression, MrkH and MrkJ, functioned similarly in *K. quasipneumoniae* as in K. pneumoniae. MrkH and MrkJ function, respectively, as the major activator and repressor of the *mrkABCDF* gene cluster in K. pneumoniae (Fig. 5A) (37, 38). To test the functions of MrkH and MrkJ in *K. quasipneumoniae*, we generated isogenic KqPF9*ΔmrkH* and KqPF9*ΔmrkJ* mutant strains (Fig. S4). We also measured *mrkH* and *mrkJ* expression in mutant and complement strains by qRT-PCR (Fig. 5B and C). To evaluate the activation and repression of *mrk* gene cluster expression by *mrkH* and *mrkJ,* respectively, we studied the expression of *mrkA*. In corroboration with observations previously reported in K. pneumoniae, *mrkA* expression was significantly decreased in the KqPF9*ΔmrkH* strain (2.9-fold) and increased in the KqPF9*ΔmrkJ* strain (2.5-fold) (Fig. 5D) (38). In both cases, the respective complement strains, namely, KqPF9*ΔmrkH*+*pmrkH* and KqPF9*ΔmrkJ*+*pmrkJ*, rescued *mrkA* expression levels (Fig. 5D). Phenotypic analysis showed that bladder epithelial cell association followed a pattern analogous to *mrkA* expression. We observed that the relative bladder epithelial cell association of KqPF9*ΔmrkH* decreased to 2.4% of that observed in wild-type KqPF9 and was rescued upon complementation in KqPF9*ΔmrkH*+*pmrkH* (Fig. 5E). Conversely, relative bladder epithelial cell association increased to 217% in KqPF9*ΔmrkJ* compared to wild-type KqPF9. Similar to *mrkA* expression, relative cell association was reduced to 19.1% in the KqPF9*ΔmrkJ*+*pmrkJ*-complemented strain compared to wild-type, which was likely due to *mrkJ* overexpression (Fig. 5E). Because regulation of type 3 fimbriae by MrkH and MrkJ has been shown to play an important role in biofilm formation in K. pneumoniae, we also assessed the role of these regulators in *K. quasipneumoniae* biofilm formation (38). The biofilm assay results followed a similar pattern as cell association (Fig. 5F). Relative biofilm formation was significantly decreased in KqPF9*ΔmrkH* to 20.6% and increased to 161% in KqPF9*ΔmrkJ*. These results mirror previously reported findings in uropathogenic K. pneumoniae strain AJ218 (38). Taken together, these results suggest that MrkH and MrkJ have similar functions in *K. quasipneumoniae* and K. pneumoniae with MrkH acting as an activator of type 3 fimbriae expression and MrkJ acting as a repressor.

### Overexpression of TOP52 but not KqPF9 *fimK* downregulates expression of type 3 fimbriae.

**FIG 6:**
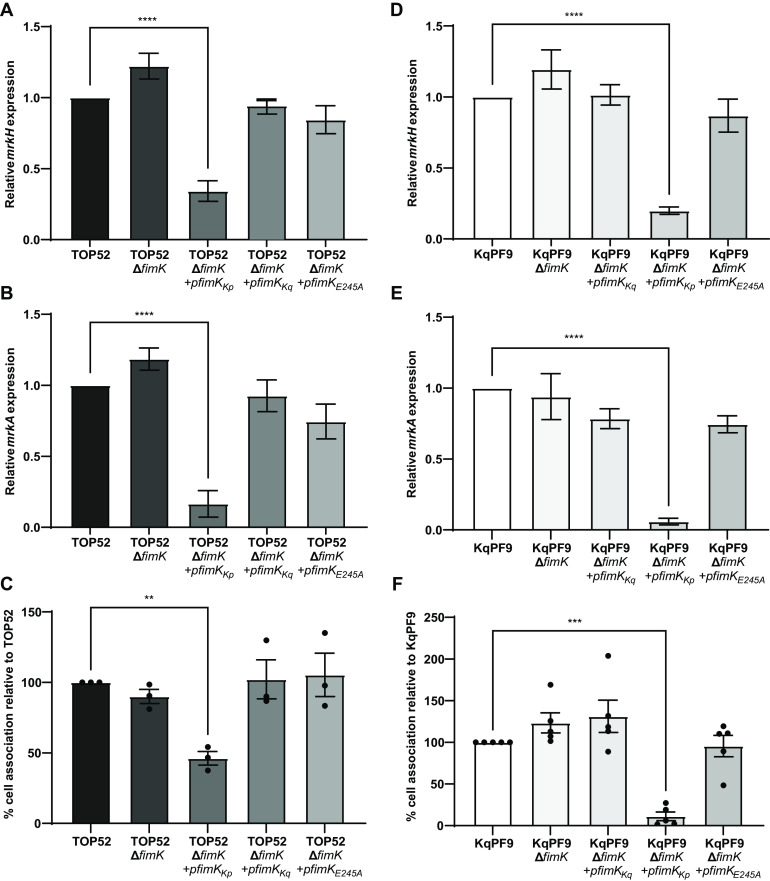
TOP52 *fimK* but not KpPF9 *fimK* reduces type 3 fimbrial expression and bladder epithelial cell attachment. (A and B) qRT-PCR analysis of *mrkH* and type 3 fimbriae (*mrkA*) gene transcription in wild-type TOP52 and respective TOP52Δ*fimK* mutant and complemented strains. The expression of each gene was normalized to *rho*, and the fold change is relative to wild type. (C) 5637 bladder epithelial cell association of TOP52 and respective TOP52Δ*fimK* and complemented strains. qRT-PCR evaluating the expression of *mrkH* and *mrkA* in wild-type KqPF9 and respective (D and E) KqPF9Δ*fimK* mutant and complement strains. *rho* Was used for normalization, and expression is relative to wild type. (F) 5637 bladder epithelial cell association of KqPF9 and KqPF9Δ*fimK* mutant and complemented strains. All experiments were performed at least in biological and technical triplicate. The error bars indicate standard error of mean. One-way ANOVA with Dunnett’s multiple-comparison test was used to evaluate significance. **, *P* < 0.01; ***, *P* < 0.005; and ****, *P* < 0.0005.

To evaluate the role of the C-terminal PDE domain of FimK in the regulation of type 3 fimbriae expression, we generated TOP52*ΔfimK* strains complemented with plasmids *pfimK_Kp_*, *pfimK_E245A_*, or *pfimK_Kq_* with *fimK_Kp_* encoding the full-length TOP52 *fimK* allele, *fimK_E245A_* encoding a PDE dead allele of TOP52 *fimK*, and *fimK_Kq_* encoding KqPF9 *fimK* allele which lacks the C-terminal PDE domain (Fig. S5) (41, 54, 55). Although no significant difference in *mrkH* or *mrkA* expression was observed between TOP52*ΔfimK* and wild-type TOP52, complementation of TOP52*ΔfimK* with TOP52 *fimK* (*pfimK_Kp_*) showed a significant reduction in *mrkH* (2.94-fold) and *mrkA* (6.25-fold) expression (Fig. 6A and B). Conversely, complementation of TOP52*ΔfimK* with *pfimK_Kq_* or *pfimK_E245A_* resulted in *mrkH* and *mrkA* expression that remained at levels comparable to wild-type TOP52 (Fig. 6A and B). Analogous to the changes in *mrkH* and *mrkA* expression, relative bladder epithelial cell association was significantly decreased to 46.14% in TOP52*ΔfimK+pfimK_Kp_* compared to wild-type TOP52 while TOP52*ΔfimK+pfimK_Kq_* and TOP52*ΔfimK+pfimK_E245A_* showed no significant difference in cell association (Fig. 6C).

Considering that only expression of the *fimK* allele containing an intact C-terminal PDE reduced type 3 fimbriae expression in TOP52, we hypothesized that its absence in KqPF9 FimK would translate to an inability to regulate type 3 fimbrial expression in *K. quasipneumoniae*. We generated an isogenic *ΔfimK* mutant of KqPF9 along with complement strains containing either plasmids *pfimK_Kq_*, *pfimK_Kp_*, or *pfimK_E245A_*. Similar to TOP52, significant decreases in *mrkH* (5.2-fold) and *mrkA* (20-fold) expression were observed in KqPF9*ΔfimK+pfimK_Kp_* compared to wild-type KqPF9 (Fig. 6D and E). However, complementation of KqPF9*ΔfimK* with the PDE-dead TOP52 *fimK* allele (*fimK_E245A_*) or KqPF9 *fimK* did not alter *mrkA* or *mrkH* expression from wild-type levels (Fig. 6D and E). Similarly, relative bladder epithelial cell association decreased to 11.2% in KqPF9*ΔfimK+pfimK_Kp_* whereas no change from wild-type KqPF9 was observed in the association of KqPF9*ΔfimK+pfimK_Kq_* and KqPF9*ΔfimK+pfimK_E245A_* (Fig. 6F). Taken together these results suggest that while the C-terminal PDE of FimK may function to coregulate type 3 fimbriae expression in TOP52, truncation of this domain in KqPF9 suggests that this activity is not conserved in *K. quasipneumoniae.*

## DISCUSSION

*K. quasipneumoniae*, previously designated as K. pneumoniae phylogroup KpII, was recently classified as a distinct species of Klebsiella (39). Previous reports indicate misidentification of *K. quasipneumoniae* as K. pneumoniae due to a lack of appropriate molecular biology tools in hospital settings (56). Considering that *K. quasipneumoniae* exhibits unique metabolic phenotypes, frequently harbors antibiotic resistance genes like ESBLs and carbapenemases, and is commonly isolated from patients with UTI, a more detailed understanding of the virulence mechanisms involved in bladder colonization of *K. quasipneumoniae* is needed (2).

Both type 1 and type 3 fimbriae have been reported to mediate host cell attachment and invasion by K. pneumoniae and are therefore crucial to infection (46, 57). However, no study has measured the expression of type 1 and type 3 fimbriae in *K. quasipneumoniae* and evaluated their respective contributions to cultured bladder epithelial cell attachment. Using isogenic mutants lacking the major subunits of type 1 and type 3 fimbriae, we show that *K. quasipneumoniae* strain KqPF9 attachment to cultured bladder epithelial cells is dependent on type 3 but not type 1 fimbriae. While cell attachment was sharply reduced in KqPF9*ΔmrkA*, no significant difference was observed between KqPF9*ΔfimA* and wild-type KqPF9. Previous reports indicate that type 1, but not type 3, fimbriae are important for K. pneumoniae bladder colonization in mice although type 3 fimbriae were found to contribute to cultured bladder epithelial cell attachment *in vitro* (19, 22). It has yet to be determined if type 3 fimbriae are required for *K. quasipneumoniae* bladder colonization in mice, an important subject for future studies.

The regulatory mechanisms controlling the expression of type 1 and type 3 fimbriae in Klebsiella spp. are likely more complex than our current understanding. Intracellular second messengers like cyclic-di-GMP play a crucial role in responding to sensory input from extracellular stimuli and coupling them to physiologic changes (58–60). Diguanylate cyclases coordinate the synthesis of cyclic-di-GMP while PDEs with conserved EAL domains are responsible for cyclic-di-GMP degradation (61–64). The type 3 fimbriae expression in Klebsiella spp. is to a large extent dependent on the transcriptional activator MrkH, a cyclic-di-GMP binding protein, and the MrkJ PDE (38). Interestingly, the FimK protein of K. pneumoniae has two domains, an N-terminal DNA binding domain and a C-terminal PDE domain that has been shown to hydrolyze cyclic-di-GMP and thereby regulate cyclic-di-GMP levels (41). It is therefore possible that K. pneumoniae FimK contributes to the regulation of type 3 fimbriae by modulating cyclic-di-GMP levels.

The observed requirement for type 3 fimbriae for *K. quasipneumoniae* cultured bladder epithelial cell association *in vitro* could be attributed to differences in expression due to differential regulation of type 1 and type 3 fimbriae. Indeed, we observed that basal expression of *mrkA* during static growth in LB and rates of *in vitro* bladder epithelial cell association were significantly higher in KqPF9 than in K. pneumoniae TOP52. Through comparative genomics of the type 1 fimbrial operons of several K. pneumoniae and *K. quasipneumoniae* strains, we discovered a premature stop codon in KqPF9 *fimK* that results in truncation of the C-terminal PDE domain and is conserved among *K. quasipneumoniae* isolates. We further demonstrated the role of the TOP52 FimK PDE in regulating type 3 fimbriae expression and bladder epithelial cell attachment *in vitro*. These results suggest that the higher basal expression of type 3 fimbriae in KqPF9 relative to TOP52 may at least be, in part, attributed to FimK PDE domain truncation. We also show that MrkH and MrkJ have conserved respective activator and repressor functions in the regulation of type 3 fimbriae in *K. quasipneumoniae.*

Interestingly, we found that deletion of *fimK* did not significantly elevate *mrkA* expression in TOP52. We hypothesize that this may be due to functional redundancy with other phosphodiesterases like MrkJ. However, it is possible that the presence of other diguanylate cyclases and phosphodiesterases in Klebsiella quasipneumoniae may contribute to the coregulation of type 1 and type 3 fimbriae in different host environments. Also, previous work has shown that FimK inhibits type 1 fimbrial expression in TOP52 and that TOP52Δ*fimK* exhibits significantly increased type 1 fimbrial expression and bladder colonization (27). The absence of the PDE domain in *fimK* of KqPF9 alongside an increase in expression of type 1 (*fimA*) and type 3 (*mrkA*) fimbriae may explain the increased biofilm formation and bladder epithelial cell association of this strain relative to TOP52. However, it is yet unclear if the increased cell association of KqPF9 strain translates to increased colonization of the urinary tract *in vivo* and if KqPF9 bladder colonization is dependent on type 1 or type 3 fimbriae.

It is also possible that additional regulatory differences exist between the two species that may contribute to differences in expression of type 1 and type 3 fimbriae. In addition to local regulators of type 1 and type 3 fimbriae encoded within the *fim* and *mrk* operons, global regulators like integration host factor (IHF), leucine regulatory proteins (LRP), and histone-like nucleoid structuring protein (H-NS) also play an important role in fimbrial regulation (65–68). In E. coli, IHF and LRP are required to maintain the phase on the orientational bias of the *fimS* switch controlling type 1 fimbriae expression while H-NS is required to maintain the phase off orientation (68). H-NS has been reported to regulate type 1 fimbriae in E. coli and K. pneumoniae and type 3 fimbriae in K. pneumoniae (65, 66, 69). H-NS regulates the phase variation of the type 1 fimbriae *fimS* region directly by binding to the region adjacent to *fimS* (70–72). H-NS also regulates type 1 fimbriae indirectly by repressing the transcription of *fimB* and *fimE* recombinases (73). In the case of type 3 fimbriae, H-NS represses the expression of *mrkH* and *mrkI* (65) and activates *mrkA* expression (74). Interestingly, cyclic-di-GMP has been reported to alleviate H-NS repression of genes involved in biofilm formation in Vibrio cholerae (75). Considering that the MrkH activator is dependent on cyclic-di-GMP, it is possible that cyclic-di-GMP activates regulators like MrkH whose binding site overlaps with binding sites of H-NS (74, 76), thereby coordinating derepression of genes involved in specific function that were originally repressed by H-NS (49, 74). Future studies should focus on disentangling these additional regulatory mechanisms in *K. quasipneumoniae.*

Taken together, the data presented here provide new insight into the regulation of type 3 fimbriae by *fimK* in K. pneumoniae as well as provide evidence for important differences in fimbrial expression and *in vitro* bladder epithelial cell attachment phenotypes between K. pneumoniae and *K. quasipneumoniae* species.

## MATERIALS AND METHODS

### Bacterial strains and culture conditions.

Bacterial strains and plasmids used in the study are listed in Table 1. KqPF9, a *K. quasipneumoniae* strain isolated from the urine of a postmenopausal woman with active, symptomatic rUTI (5); TOP52, a K. pneumoniae strain isolated from the urine of a woman with acute cystitis; and UTI89, a UPEC acute cystitis isolate, were used in the study. The complete KqPF9 genome has been described previously and is available on GenBank through BioProject PRJNA683049 (5). Bacteria were grown in Luria Bertani (LB) media overnight at 37°C in static conditions. For RNA extractions in quantitative reverse transcriptase PCR (qRT-PCR) assays, overnight cultures were subcultured at a starting optical density at 600 nm (OD_600_) of 0.01 and then incubated for 6 h until reaching the midlog phase (OD_600_ = 0.4 – 0.6) induced with 0.2% l-arabinose (*mrkA* genotypes) or 0.0125% l-arabinose (*fimA*, *mrkH*, and *mrkJ* genotypes) at 37°C in LB media in an incubator at static conditions. For negative staining electron microscopy experiments, bacteria were grown for 8 h at 37°C in static conditions with 0.2% l-arabinose for induction of complementation in the respective strains.

**TABLE 1:** Strains and plasmids used in this study

Strains and plasmids	Description	Source
PF9	*K. quasipneumoniae* cystitis isolate	This study
TOP52 1721	K. pneumoniae cystitis isolate	Rosen et al. (27)
UTI89	E. coli cystitis isolate	Mulvey et al. (86)
KqPF9*ΔmrkA*	Knockout of *mrkA* in PF9	This study
KqPF9*ΔfimA*	Knockout of *fimA* in PF9	This study
KqPF9*ΔmrkAΔfimA*	Knockout of *mrkA* and *fimA* in PF9	This study
KqPF9*ΔmrkH*	Knockout of *mrkH* in PF9	This study
KqPF9*ΔmrkJ*	Knockout of *mrkJ* in PF9	This study
KqPF9*ΔfimK*	Knockout of *fimK* in PF9	This study
TOP52*ΔfimK*	Knockout of *fimK* in TOP52	This study
*pBAD-e*mpty vector	Empty vector (EV), arabinose inducible, Kan^R^	Guzman et al., 1995 (79)
*pmrkgc*	Plasmid expressing *mrk* gene cluster	This study
*pfimgc*	Plasmid expressing *fim* gene cluster	This study
*pmrkH*	Plasmid expressing *mrkH*	This study
*pmrkJ*	Plasmid expressing *mrkJ*	This study
*pfimK_Kp_*	Plasmid expressing WT TOP52*fimK*	This study
*pfimK_Kq_*	Plasmid expressing WT KqPF9 *fimK*	This study
*pfimK_E245A_*	Plasmid expressing mutant TOP52 *fimK*-*AIL*	This study

### Bacterial cell association and gentamicin protection (invasion) assays.

Cell association and invasion assays were performed as previously described (44). Urinary bladder epithelial 5637 cells (ATCC no. HTB-9) were cultivated in RPMI (Sigma) supplemented with 10% FBS (ThermoFisher), 1% penicillin-streptomycin (Gibco), and 1% glutamax (Gibco) overnight at 37°C and 5% CO_2_. The next day, cells were washed with phosphate-buffered saline (PBS) and incubated in RPMI without antibiotics. Cells were infected at a multiplicity of infection (MOI) of 10. Bacterial contact with host cells was synchronized by centrifugation of plates at 600 × *g* for 5 min. For association assays, 2 h postinfection cells were washed in 1× PBS and then harvested and lysed with 0.3% Triton X-100. Cells in the input wells, representing total intra- and extracellular bacteria, were not washed and directly lysed with Triton X-100. For gentamicin protection (invasion) assays, RPMI was replaced with RPMI containing 100 μg/ml gentamicin 2 h postinfection and incubated for an additional 2 h before harvesting as described above. Association and invasion frequencies were calculated as a ratio of the number of bacteria recovered from washed lysates to the total number of bacteria present in the input (unwashed) wells. All cell association and invasion assays were performed at least in biological triplicate. For each biological replicate, a minimum of three technical replicates were evaluated.

### Generation of *K. quasipneumoniae* and K. pneumoniae gene deletion mutants.

Type 1 fimbriae knockout, type 3 fimbriae knockout, or both type 1 and type 3 fimbriae knockout mutants of *K. quasipneumoniae* were generated by targeted gene deletion of genes encoding the major fimbrial subunits, *fimA* and *mrkA*, respectively (36, 46). All gene deletions were performed using Lambda Red Recombinase system as previously described with respective primers listed in Table S2 (77). The plasmid pKD4 served as the template to amplify the kanamycin cassette and flanking FRT sites (78) to generate knockout cassettes respective to each gene. The Lambda Red Recombinase system was expressed from pACBSR-Hyg, which carries *beta*, *gam*, and *exo* under the control of an arabinose inducible promoter. pFLP-Hyg, which expresses the FLP recombinase, was used to excise the kanamycin cassette via the FRT sites. PCR was performed using flanking primers mentioned in Table S2 to screen candidate colonies using Dreamtaq Master Mix (ThermoFisher). PCR products were analyzed by agarose gel electrophoresis and Sanger sequencing (Genewiz) to confirm gene deletion.

### Generation of complementation plasmids.

For the construction of complementation plasmids, genes were amplified from genomic DNA by PCR using Dreamtaq Mastermix (ThermoFisher) or PHUsion polymerase for PCR products >2,000 bp (NEB) with respective primers as provided in Table S2. For all plasmids, NcoI and HindIII restriction sites were used except for plasmids encoding *mrk* and *fim* gene clusters where EcoRI and XhoI sites were used for ligation into pBAD33 (Kan^R^) version A (79). For the generation of the plasmid encoding the AIL mutant of *fimK_E245A_*, site-directed mutagenesis was performed on *pfimK_Kp_* with the mutagenesis primers listed in Table S2 followed by DpnI digestion. All plasmids were sequence verified by Sanger sequencing before transformation into respective isogenic mutant strains.

### Bacterial transformation.

Transformation of Klebsiella strains KqPF9 and TOP52 was performed by electroporation of respective electrocompetent cells in a 0.1-cm gap-width cuvette at 2.5 kV using a micropulser electroporator (Bio-Rad) and selected on LB agar plates with 100 μg/ml kanamycin for Lambda Red Recombineering protocols and selection of complementation plasmids (77). Chemical transformation was used during subcloning to introduce complementation plasmid constructs into E. coli DH5α (79).

### RNA extraction, cDNA synthesis, and qRT-PCR.

Total RNA was extracted from respective statically growing *K*qPF9 and TOP52 at midlog phase (OD_600_ = 0.5 to 0.6) using the Qiagen RNeasy Plus kit by following the manufacturer’s instructions. RNA concentration and quality were analyzed by NanoDrop (Thermo). RNA with A_260/280_ ratios between 2.0 and 2.20 was used for cDNA synthesis using the Qscript cDNA kit (Quantabio). Following cDNA synthesis, qPCR was performed using PerfeCTa SYBR green FastMix (Quantabio) and respective primer pairs listed in Table S3. The expression of the gene of interest was normalized to the expression of *rho* (80). Relative expression was determined by the quantification cycle (ΔΔ*Cq*) method (81, 82). qRT-PCR was performed in biological and technical triplicates.

### Static biofilm assays.

Static biofilm assays were performed as described previously (83). Bacteria were grown overnight in LB at 37°C under static conditions. Bacteria were normalized to an OD_600_ of 0.1 in LB broth and seeded into round bottom 96-well plates (Corning). Then, 0.2% l-arabinose was added for induction of complementation plasmids. Plates were incubated for 23 h at 37°C in static conditions and then washed (2×) with sterile water and dried for 25 min. Biofilms were fixed with methanol, stained with a 0.1% (wt/vol) crystal violet solution (Sigma), and washed (2×) with sterile water. The bound dye was solubilized with 30% acetic acid and the OD_585_ was measured. Each experiment was performed at least three times in technical triplicate.

### Yeast agglutination assays.

Bacterial strains were grown overnight at 37°C in Luria Bertani (LB) media at static conditions. Cultures were normalized to an OD_600_ of 0.5 and centrifuged at 5,000 rpm for 5 min. Bacterial pellets were then resuspended in 1× sterile PBS or PBS + 2.5% d-mannose solutions. The Saccharomyces cerevisiae strain L40 was grown overnight at 27°C in yeast peptone dextrose media at 225 rpm. Yeast cells were normalized to an OD_600_ of 0.5, pelleted at 5,000 rpm, and resuspended in 1× sterile PBS. Bacterial and yeast suspensions were then mixed in a 1:1 ratio on a glass slide and gently rotated until agglutination became visible and imaged under a light microscope (Zeiss). Images were obtained using XCAM1080PHD and SEBA view software.

### Human red blood cell isolation and tannic acid treatment.

Red blood cells (RBCs) were isolated via centrifugation of fresh whole human blood (Fisher). Briefly, whole blood was centrifuged at 1,000 × *g* for 10 min at 4°C, the supernatant was aspirated, and the pellet was resuspended in a 2× volume of RBC isolation buffer (21.0 mM Tris, 4.7 mM KCl, 2.0 mM CaCl_2_, 140.5 mM NaCl, 1.2 mM MgSO_4_, 5.5 mM glucose, and 0.5% bovine albumin fraction V). This procedure was then repeated for a total of three washes. Isolated RBCs were then diluted in sterile PBS to make a 10% vol/vol solution. For tannic acid treatment of the RBCs, tannic acid was added to a final concentration of 0.1% and RBCs were incubated for 10 min at 37°C. The cells were then washed 1× in sterile 1× PBS and resuspended in sterile 1× PBS to make a 5% solution.

### Tanned human RBC agglutination assay.

Agglutination assays using tannic acid-treated human RBCs were performed as described previously (25). Briefly, KqPF9 and UTI89 were grown overnight statically at 37°C in Luria Bertani (LB) broth. Overnight cultures were normalized to an OD_600_ of 0.5 with sterile 1× PBS. Once normalized, 1 ml of culture was pelleted and then resuspended in 1 ml of sterile PBS. Tanned human RBCs and bacterial solutions were then added at a 1:1 ratio to the surface of a glass slide and gently rocked until mixed. Slides were observed and agglutination was scored as present (+) or absent (−) after 10 min. Tanned human RBCs added to an equal volume of 1× PBS were used as an additional negative control.

### Multiple sequence alignments.

The *fimK* genes of 10 different K. pneumoniae and *K. quasipneumoniae* strains that were previously reported in the NCBI database were compiled. The *fimK* sequences of three *K. quasipneumoniae* isolates with sequence IDs CP065841.1 (PF9), CP065838.1 (PF26), and CP065846.1 (PF42) whose whole-genome sequencing was performed and previously reported were also used for alignment (5). Following collation of the translated *fimK* sequences, their amino acid sequence alignment was performed using Muscle software with default parameters (52, 53). The alignments were then exported to NCBI’s multiple sequence aligner for further analysis.

### Phase assay of the *fimS* invertible element of *fim* operon.

The *fimS* phase assays were performed similarly as described previously (84). KqPF9 and TOP52 isolates were grown overnight at 37°C in static conditions and harvested by centrifugation, and their genomic DNA was extracted using the Easy-DNA kit (Invitrogen). PCR primers were designed as listed in Table S2 to amplify nucleotide segments 5′ and 3′ to the respective *fimS* regions (Fig. S3C). The “on” phase generated a PCR product of size 905 bp while the “off” phase generated a product of size 356 bp. Based on the size of the PCR products as analyzed by 1% agarose gel electrophoresis, the corresponding “on” or “off” phase of the *fimS* switch was determined.

### Negative stain electron microscopy.

Negative staining and electron microscopy were performed as previously described (85). Briefly, a Formvar-coated carbon-reinforced copper grid was placed with the film side down, on a droplet of a bacterial suspension for 2 min. Filter paper was used to remove excess liquid and the grid was stained for 30 s on droplets of 1.25% phosphotungstic acid (pH 6.5). Electron microscopy was performed with a JEOL 1400+ transmission electron microscope using Gatan Microscopy Suite (GMS) software.

### Statistical analysis.

All statistical analysis was performed using GraphPad Prism (9.1.0). For pairwise comparisons, significance was evaluated by paired two-tailed Student’s *t* test. For multiple comparisons, significance was evaluated using one-way ANOVA with Dunnett’s multiple-comparison test. For all tests, significance was as follows: *, *P ≤ *0.05; **, *P ≤ *0.01; ***, *P ≤ *0.001; ****, *P ≤ *0.0001.
