# Supplementary material for: Conserved FimK Truncation Coincides with Increased Expression of Type 3 Fimbriae and Cultured Bladder Epithelial Cell Association in Klebsiella quasipneumoniae
Source: J Bacteriol. 2022 Aug 25;204(9):e00172-22. doi: 10.1128/jb.00172-22 (PMC9487511; doi:10.1128/jb.00172-22)
Supplement: Supplemental file 1 — Fig. S1 to S5 and Tables S1 to S3. Download jb.00172-22-s0001.pdf, PDF file, 0.5 MB [file jb.00172-22-s0001.pdf]

Supplemental material

Conserved FimK truncation coincides with increased expression of type 3 fimbriae and bladder epithelial cell association in *Klebsiella quasipneumoniae*

Sundharamani Venkitapathi, Yalini H. Wijesundara, Samuel A. Cornelius, Fabian C. Herbert, Jeremiah J. Gassensmith, Philippe E. Zimmern, and Nicole J. De Nisco

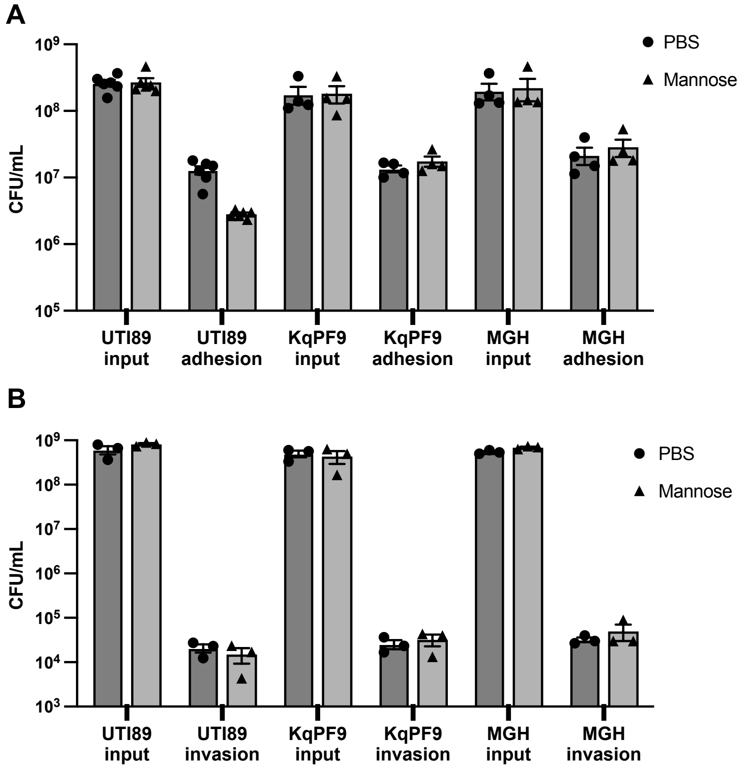

Figure S1. Colony forming unit counts for mannose adhesion and invasion assays. A) Colony forming unit (CFUs) data used to generate % relative association plot in Figure 1C. **B)** CFU data used to generate % relative invasion plot in Figure 1D and invasion plots (B) in Figures 1C and 1D, respectively. Circles (●) represent PBS-treated controls and triangles (▲) represented bacteria treated with 2.5% D-mannose. Error bars indicate standard error of the mean.

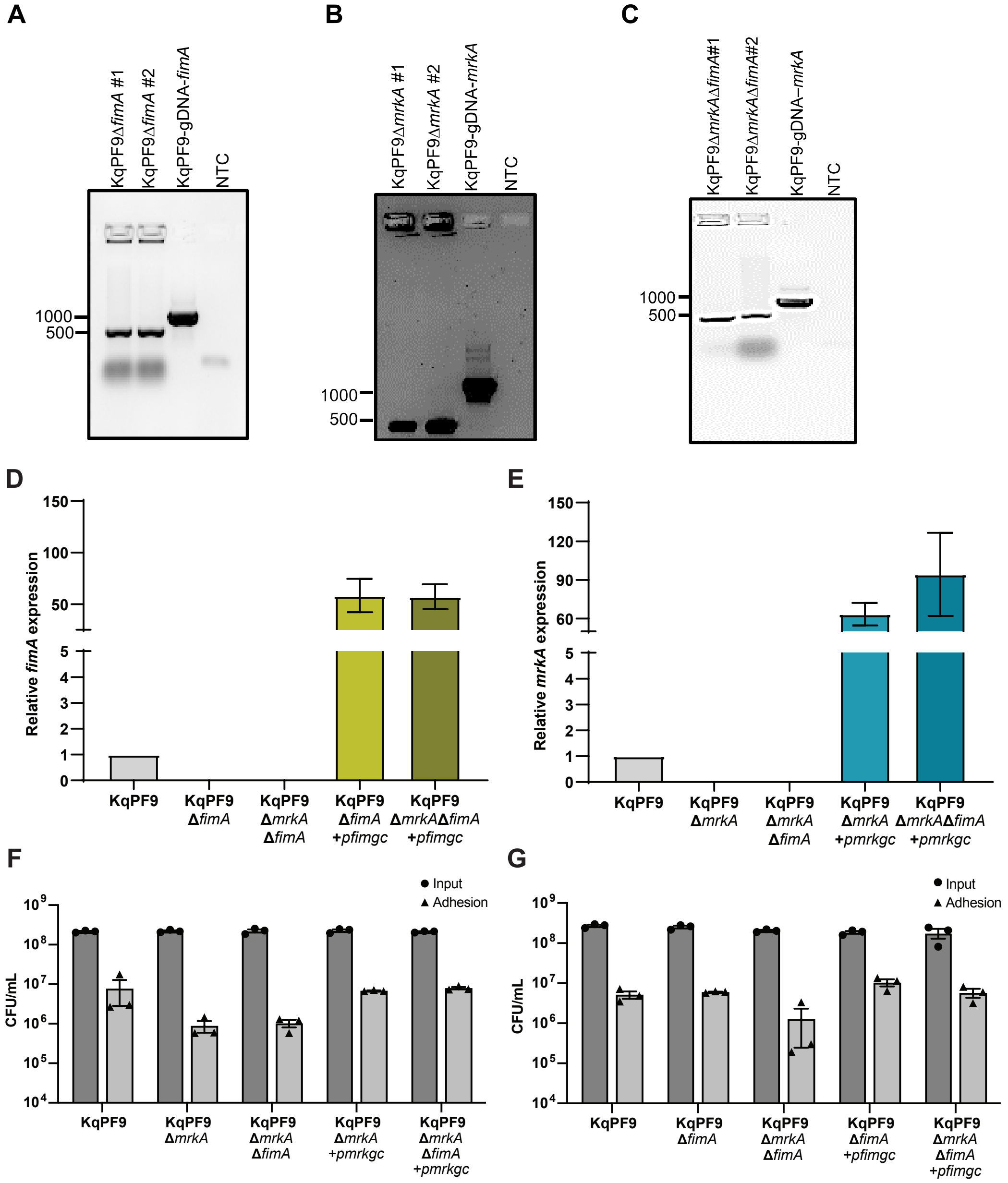

Figure S2. KqPF9 type 1 and type 3 fimbriae knockout and complement confirmation and CFU adhesion assay data. Images of the PCR products indicating successful gene deletion in **A)** type 1 (KqPF9Δ*fimA*), **B)** type 3 (KqPF9Δ*mrkA*) or **C)** type 1 and type 3 fimbriae (KqPF9Δ*mrkA*Δ*fimA*) double mutants of KqPF9 resolved on an 1% agarose gel. KqPF9 was used as positive control and water as a no template control (NTC). **D)** Quantitative reverse transcriptase PCR (qRT-PCR) analysis type 1 fimbriae (*fimA*) and **E)** type 3 (*mrkA*) fimbriae expression in KqPF9 and respective isogenic mutant and complement strains. *fimgc* indicates the *fim* gene cluster (*fimAICDFGHK*). *mrkgc* indicates the *mrk* gene cluster (*mrkABCDF*). **F)**

Colony forming unit (CFU) data used to produce relative %adhesion plots in Figure 2B. **G)** CFU data used to plot relative %adhesion in Figure 2C. Circles (•) represent input CFUs and triangles (▲) represent CFUs of associated bacteria. For qRT-PCR, expression of each gene was normalized to expression of *rho* and fold change is expressed relative to wild-type. Error bars indicate standard error of the mean.

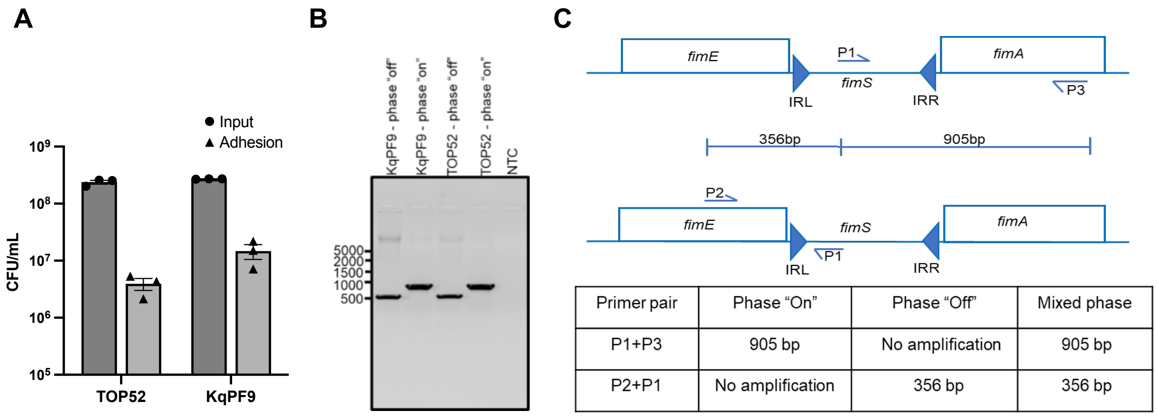

Figure S3. Association assay CFU data and *fimS* phase assay. A) Colony forming unit (CFU) data used to plot relative % adhesion data in Figure 3C. Circles (•) represent input CFUs and triangles (▲) represent CFUs of associated bacteria. **B)** PCR amplification products of genomic DNA extracted from overnight static cultures of wildtype KqPF9 and TOP52 using either phase "on" or phase "off" primer sets" resolved on a 1% agarose gel. Water was used as a no template control (NTC). Banding patterns indicate *fimS* exists in both "on" and "off" orientations in KqPF9 and TOP52 during static culture. **C)** Schematic representation of expected band sizes of the "on" and "off" phases of the "*fimS*" invertible DNA element. The sequences of respective primers P1, P2 and P3 are indicated in Table S3.

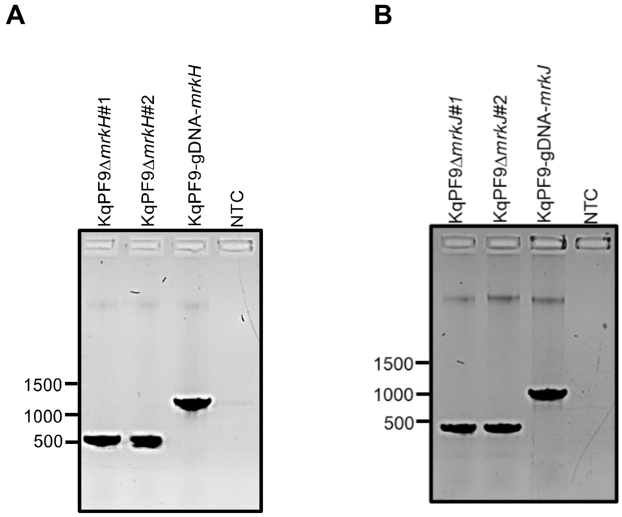

Figure S4. Generation of isogenic *mrkH* and *mrkJ* mutants of KqPF9. Verification PCR of isogenic **A)** *mrkH* and **B)** *mrkJ* mutants of KqPF9 resolved in an 1% agarose gel. The genomic DNA of KqPF9 was used as positive control for wildtype PCR product size and water was used as no template control (NTC).

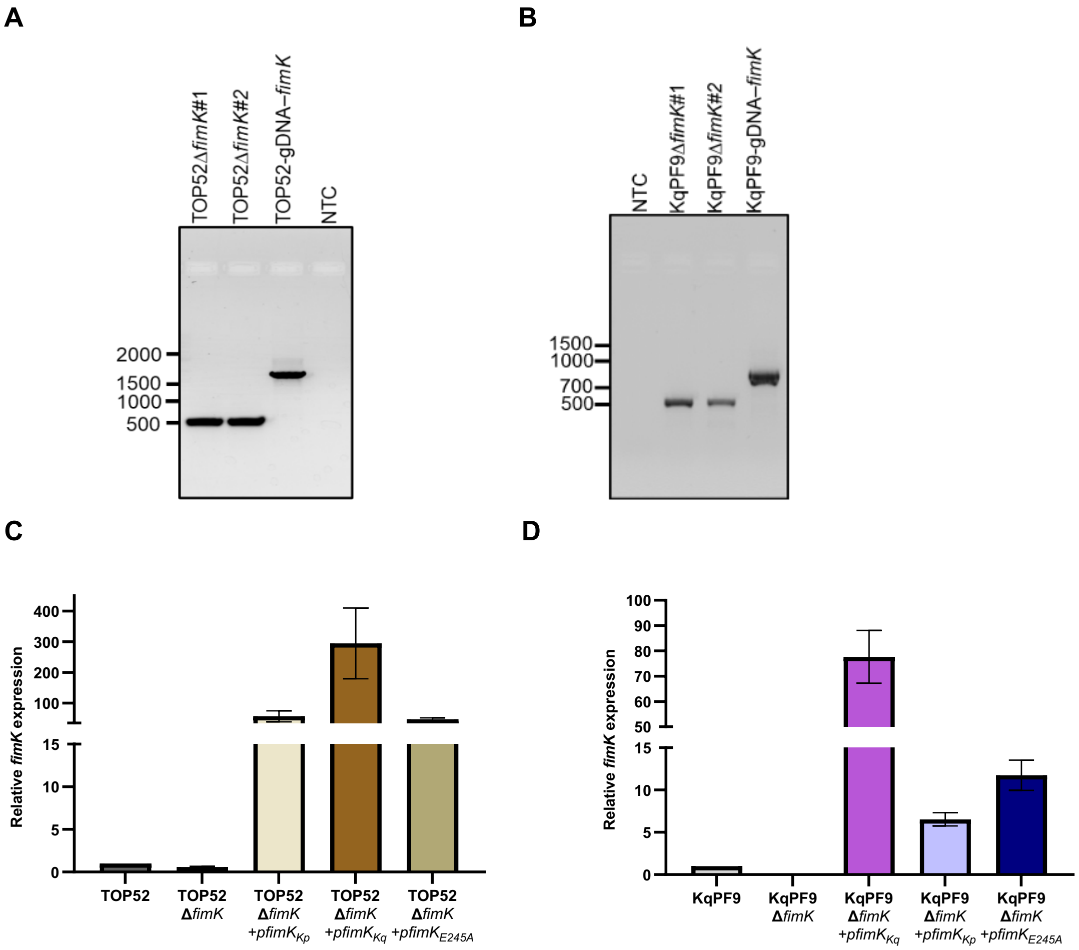

Figure S5. Verification of isogenic *fimK* mutant and complement strains of TOP52 and KqPF9. PCR confirmation of *fimK* knockouts in **A)** TOP52 and **B)** KqPF9 is shown as resolved through 1% agarose gel. Genomic DNA of respective wildtype strains of TOP52 and KqPF9 were used as controls to verify the PCR product size and water as a no template control (NTC). qRT-PCR analysis of *fimK* expression in **C)** TOP52 and **D)** KqPF9 and isogenic *fimK* mutants and complement strains carried out in biological and technical triplicate. *rho* was used for normalization and relative expression is in respect to wildtype. The bar plot depicts the mean and error bars indicate SEM.

Strain	Yeast Agglutination Score		Tanned H-RBC Agglutination Score
	PBS	2.5% Mannose	
UTI89	+++	-	-
KqPF9	+++	++	+
KqPF9Δ*mrkA*	+	-	NT
KqPF9Δ*fimA*	++	+	NT
KqPF9Δ*mrkA*Δ*fimA*	-	-	NT

Table S1. KqPF9 yeast and tanned human red blood cell agglutination patterns suggest expression of type 3 fimbriae. Scoring of the yeast and tanned human red blood cell (H-RBC) agglutination patterns by UTI89 and the KqPF9 wild-type and mutant strains used in this study. Agglutination of yeast was scored as high (+++, 67-100% agglutination), medium (++, 33-66% agglutination), low (+, <33% agglutination), and absent (-). Yeast agglutinations were performed in the presence and absence (PBS) of 2.5% D-mannose to determine dependence on type 1 fimbriae. H-RBC agglutination was scored as present (+) or absent (-) and was measured to confirm expression of type 3 fimbriae in KqPF9. Scoring represents the averages of three independent experiments. NT = not tested.

Table S2. Primer sequences used to generate and verify isogenic mutant strains of KqPF9 and TOP52.

Primer name	Primer use	Primer Sequence (5'- 3')
PF9-*mrkA*-ko-f	Knockout cassette for *mrkA* deletion in PF9	TATGCGAATTCACAGTGTGCTCATTGATTCGTAA TTCACTCTGACAAGGAAATGGCAATGTGTGTAG GCTGGAGCTGCTTC
PF9-*mrkA*-ko-r	Knockout cassette for *mrkA* deletion in PF9	CGCTTTATTATTGTTATTAACTGCCCCATCGCGG GGCAGTTTTATTTTCTGACGGAATTAGCTGACAT GGGAATTAGCCATGG
PF9-*fimA*-ko-f	Knockout cassette for *fimA* deletion in PF9	AGGCACAACGGCTGCCAATCCGGTTCGTTATTT CGACATCGTTCAAAGGAAAACAGTATGTGTGTA GGCTGGAGCTGCTTC
PF9-*fimA*-ko-r	Knockout cassette for *fimA* deletion in PF9	TGCAAAATTAAGCGCGGGCCCTCCTGGCCCGGA TGGCTTCCTTGCCTGATGTTTGCCTTAGCTGACA TGGGAATTAGCCATGG
PF9-*mrkH*-ko-f	Knockout cassette for *mrkH* deletion in PF9	TGTTGCTATTGCTATAAGAAAAATCAAACGCCTC ACGACAACTATTTACAAGGGATGCACTGTGTAG GCTGGAGCTGCTTC
PF9-*mrkH*-ko-r	Knockout cassette for *mrkH* deletion in PF9	GATAGATTGAGTGACCAATGAGATTATCATTGGT GTACAGCAATATACTTTCCAAGGGTAGCTGACAT GGGAATTAGCCATGG

PF9-*mrkJ*-ko-f	Knockout cassette for *mrkJ* deletion in PF9	GGTAGCCTATAATTAACCTATCCTCTGCTTATTG TCTGACTAACCTCGTGAAGAGGGATATGTGTAG GCTGGAGCTGCTTC
PF9-*mrkJ*-ko-r	Knockout cassette for *mrkJ* deletion in PF9	GCAACGTGGGAATTAGTTTATAATTAAGCGGGA CGAAAAAGCCGGGCAAGCCCGGCTTTTGCTGAC ATGGGAATTAGCCATGG
PF9-*fimK*-ko-f	Knockout cassette for *fimK* deletion in PF9	GGCCAGGTCACCGCCGGCAACGTGCAGTCGAT CATCGGCATTACCTTTGTCTATCAATGATGTGTA GGCTGGAGCTGCTTC
PF9-*fimK*-ko-r	Knockout cassette for *fimK* deletion in PF9	CGGCACCGGTGTAAACCGGTGCGCTTTTCTCTC GCCAGCGAATCCACGCCTTTAGTCACTCATCGC TTCCCCGCTGACATGGGAATTAGCCATGG
TOP52-*fimK*-ko-f	Knockout cassette for *fimK* deletion in TOP52	GGCCAGGTTACCGCCGGCAACGTGCAGTCGAT CATCGGCATCACCTTTGTCTATCAATGATGTGTA GGCTGGAGCTGCTTC
TOP52-*fimK*-ko-r	Knockout cassette for *fimK* deletion in TOP52	GACGATATTCGCGCATGACGTACCGGCACCGGT GCTAACCGGTGCGCTTTTCTCGCACCCGCTGAC ATGGGAATTAGCCATGG
mrkA-v1-for	Verify *mrkA* deletion	GCATTCTTTGACGCCGATAG
mrkA-v1-rev	Verify *mrkA* deletion	CCTGGATAAATAAAGCGGGTA
fimA-v1-for	Verify *fimA* deletion	CCACATTAAACAGATTTTAATACCG
fimA-v1-rev	Verify *fimA* deletion	CGTCGTGAAATCGGTTACTG
K1-rev	Kanamycin cassette	CAGTCATAGCCGAATAGCCT
K2-for	Kanamycin cassette	GGTGCCCTGAATGAACTGC
Kt-rev	Kanamycin cassette	GGCCACAGTCGATGAATC
mrkH-v1-for	Verify *mrkH* deletion	TCCCTCCTCAATATTTGCCTG
mrkH-v1-rev	Verify *mrkH* deletion	GATTCTGATGGCAGAAATATCCT
mrkJ-v1-for	Verify *mrkJ* deletion	TTGCCGCCCTGCTCGG
mrkJ-v1-rev	Verify *mrkJ* deletion	GTTTTTACTGACGGCGGTCG
PF9-*fimK*-v1-for	Verify *fimK* deletion	AACGCGATCTTCACTAAC
PF9-*fimK*-v1-for	Verify *fimK* deletion	TGGTGGAAAAAATGCGCC
TOP52-*fimK*-v1-f	Verify *fimK* deletion	AACAGCACGGTCTCGCT
TOP52-*fimK*-v1-r	Verify *fimK* deletion	GATGGAAATACTGGAAGGG

Table S3. Primer sequences used for generation of gene complements, qRT-PCR and *fimS* phase assay.

**Primer name**	**Primer use**	**Primer sequence (5'- 3')**
mrkABCDF-XhoI-f	Generation of p*mrkABCDEF*	GATCCTCGAGCATGAAAAAGGTTCTTCTCTCTGCA
mrkABCDF-EcoRI-r	Generation of p*mrkABCDEF*	GATCGAATTCTTAATTATAAACTAATTCCCACGTTGC
fimAICDFGHK-XhoI-f	Generation of p*fimAICDFGHK*	GATCCTCGAGCATGAAAATCAAAACACTGGCAATG
fimAICDFGHK-EcoRI-r	Generation of p*fimAICDFGHK*	GATCGAATTCTCATGCCCGGACAAACGC
PF9-*fimK*-NcoI-f	Generation of p$fimK_{KqPF9}$	GATCCCATGGCCGAGTACATCCTTTCT
PF9-*fimK*-HindIII-f	Generation of p$fimK_{KqPF9}$	GATCAAGCTTTCATGCCCGGACAAACGC
TOP52-*fimK*-NcoI-f	Generation of *p*fimK_{TOP52}	GATCCCATG GCCGATTATATCCTCTCGC
TOP52-*fimK*-HindIII-r	Generation of *p*fimK_{TOP52}	GATCAAGCTTTCAACGTTTCGCCGGATCGC
TOP52-*fimK*-AIL-f	Generation of *p*$\mathrm{fimK}_{TOP52\text{-}AIL}$	TACAGGGGGTGGCGATCCTGATCCG
TOP52-*fimK*-AIL-r	Generation of *p*$\mathrm{fimK}_{TOP52\text{-}AIL}$	CGGATCAGGATCGCCACCCCCTGTA
mrkH-NcoI-f	Generation of p*mrkH*	GATCCCATGGCAGAGGGAACGATAAAGA
mrkH-HindIII-r	Generation of p*mrkH*	GATCAAGCTTGATTCTCTTTTTTCGCTTGGCTT
mrkJ-NcoI-f	Generation of p*mrkH*	GATCCCATGGACACTAAAATATTCGAAGACAA
mrkJ-HindIII-r	Generation of p*mrkH*	GATCAAGCTTTATGCCAATATCGTCGGCAAC
PF9-*mrkA*-f	qRT-PCR	GTTACCGATGTATCCTGTAC
PF9-*mrkA*-r	qRT-PCR	GGCAGTTAGAGACGTCAA
PF9-*fimA*-f	qRT-PCR	ATGATTGTTGTGTCAGCCCT
PF9-*fimA*-r	qRT-PCR	CCCAACTGGACGGTTTGA
PF9-*fimK*-f	qRT-PCR	GGTTGAGCCAGCTGATG
PF9-*fimK*-r	qRT-PCR	GTGGTGAGCATCCACAG
TOP52-*fimK*-f	qRT-PCR	GGTTGAGCCAGCTGATG
TOP52-*fimK*-r	qRT-PCR	GTGGTGAGCATCCACAG
PF9-*mrkH*-f	qRT-PCR	ATAAAATTCGCTTTCTCCTGCAT
PF9-*mrkH*-r	qRT-PCR	TAAACGAAAGCGGGGATCG
TOP52-*mrkH*-f	qRT-PCR	ATAAAATTCGCTTTCTCCTGCAT
TOP52-*mrkH*-r	qRT-PCR	TAAACGAAAGCGGGGATCG
PF9-*rho*-f	qRT-PCR	AACTACGACAAGCCGGAAAA
PF9-*rho*-r	qRT-PCR	ACCGTTACCACGCTCCATA
TOP52-*rho*-f	qRT-PCR	AACTACGACAAGCCGGAAAA
TOP52-*rho*-r	qRT-PCR	ACCGTTACCACGCTCCATA
PF9/TOP52-fimE-f (P2)	*fimS* phase "off	GCAGGCGTATCGTATTATTCG
PF9/TOP52-fimS-f (P1)	*fimS* phase "off"	TGTTTTGACATATTTTGCAACTCAC
PF9/TOP52-fimA-r (P3)	*fimS* phase	CGTAGTAACGTGCCTGGAAC
